# Direct quantitative detection of Doc2b-induced hemifusion in optically trapped membranes

**DOI:** 10.1038/ncomms9387

**Published:** 2015-09-23

**Authors:** Ineke Brouwer, Asiya Giniatullina, Niels Laurens, Jan R. T. van Weering, Dirk Bald, Gijs J. L. Wuite, Alexander J. Groffen

**Affiliations:** 1 Department of Physics and Astronomy, LaserLab, VU University, De Boelelaan 1085, 1081 HV Amsterdam, The Netherlands; 2 Department of Functional Genomics and Clinical Genetics, CNCR, NCA, VU University and VU Medical Center, De Boelelaan 1085, 1081 HV Amsterdam, The Netherlands; 3 Department of Molecular Cell Biology, VU University, De Boelelaan 1085, 1081 HV Amsterdam, The Netherlands

## Abstract

Ca^2+^-sensor proteins control the secretion of many neuroendocrine substances. Calcium-secretion coupling may involve several mechanisms. First, Ca^2+^-dependent association of their tandem C2 domains with phosphatidylserine may induce membrane curvature and thereby enhance fusion. Second, their association with SNARE complexes may inhibit membrane fusion in the absence of a Ca^2+^ trigger. Here we present a method using two optically trapped beads coated with SNARE-free synthetic membranes to elucidate the direct role of the C2AB domain of the soluble Ca^2+^-sensor Doc2b. Contacting membranes are often coupled by a Doc2b-coated membrane stalk that resists forces up to 600 pN upon bead separation. Stalk formation depends strictly on Ca^2+^ and phosphatidylserine. Real-time fluorescence imaging shows phospholipid but not content mixing, indicating membrane hemifusion. Thus, Doc2b acts directly on membranes and stabilizes the hemifusion intermediate in this cell-free system. In living cells, this mechanism may co-occur with progressive SNARE complex assembly, together defining Ca^2+^-secretion coupling.

## Abstract (web summary)

Membrane fusion in cells is triggered by an increase in Ca^2+^ and involves SNARE complexes and calcium-sensing proteins, but the mechanism underlying the Ca^2+^-sensors' role in fusion remains unclear. Here the authors show *in vitro* that the Ca^2+^-sensor Doc2b acts directly on membranes and induces a hemifusion intermediate in the presence of calcium.

## Graphical abstract

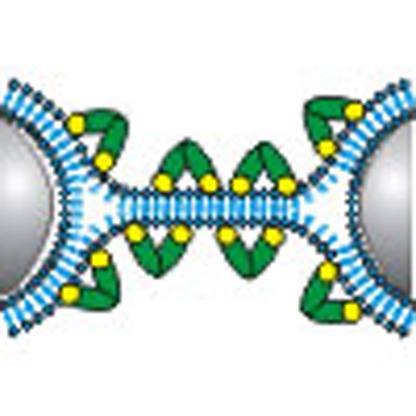

Many biological functions depend on regulated secretion. Secretory events proceed through a hemifusion intermediate in which only the proximal leaflets of the vesicle and plasma membrane have fused, as evidenced by electrophysiological measurements^1^, assays based on phospholipid versus content mixing^2^,^3^,^4^ or on the mixing of phospholipids from the outer and inner membrane leaflet^5^,^6^. A widely used mechanistic model postulates the occurrence of an hourglass-shaped intermediate or stalk structure^7^. Such structures were indeed observed by X-ray diffraction in preparations of multiple parallel bilayers^8^. Membrane fusion in living cells essentially requires the gradual assembly of the SNARE complex that progresses from a *trans*- to a *cis*-conformation^9^,^10^,^11^. The precise timing of secretory events is regulated by signalling pathways that differ between tissues, but almost always converge upon the generation of an intracellular Ca^2+^ signal. These Ca^2+^ ions activate C2 domain-containing proteins such as members of the Synaptotagmin, Doc2 and Ferlin families^12^, which interact with membranes and SNARE proteins to trigger secretion^13^.

Previous work has suggested different roles for Ca^2+^-sensors in secretion coupling. Genetic ablation of Synaptotagmin-1 (Syt1)—a membrane-anchored protein that causes mixed effects on neurotransmitter release—severely impairs fast Ca^2+^-induced secretion^14^,^15^. The direct interaction of Syt1's cytosolic C2 domains with phosphatidylserine (PS) prompted the idea that the Ca^2+^ sensor could directly act in membrane remodelling^16^. In support of this idea, the tandem C2 domains of Syt1 can induce local curvature by shallow insertion of these domains into the lipid bilayer, and this membrane-bending activity correlates with its activity in enhancing the fusion of SNARE liposomes^17^,^18^. On the other hand, the lack of Syt1 also causes increased rates of spontaneous release rate in resting synapses^14^. These observations suggested that the sensor could arrest fusion at a late stage in the secretion process until a Ca^2+^ trigger relieves this inhibitory activity^19^. Mechanistically, such an inhibitory activity was proposed to involve binding of Syt1 to the SNARE complex, thereby preventing fusion to proceed in the absence of a triggering Ca^2+^ signal (often referred to as ‘clamping')^20^.

The fusion-enhancing and clamping mechanisms are not mutually exclusive: it is possible that a sensor both inhibits membrane fusion at low Ca^2+^ and enhances it after Ca^2+^ elevation^13^. In some experimental conditions, both activities have been separated^21^,^22^. For example, in Synaptotagmin-2, a mutation of the SNARE-binding polylysine motif caused a loss of spontaneous release inhibition, whereas the fast Ca^2+^-evoked secretory response was largely normal^22^. Vice versa, mutation of a Ca^2+^-binding aspartate D364N at the membrane-inserting interface impaired the fast secretory response but left spontaneous release unaffected^22^.

Despite many elegant efforts to resolve the precise mechanism, it remains a challenge to investigate the role of the sensor–SNARE and sensor–membrane interactions by mutagenesis because the sensor's residues that mediate SNARE binding also mediate membrane binding^23^,^24^. To address this issue, we established a new method combining real-time fluorescence imaging, microfluidics and force spectroscopy to study how the Ca^2+^ sensor Doc2b interacts with two opposing membranes. We focused on the C2AB fragment of Doc2b (referred to as Doc2b in this study) for several reasons. First, soluble C2AB fragments of Doc2b and Syt1 are sufficient for Ca^2+^-dependent membrane fusion in cell-free assays^17^,^18^,^25^. In Syt1 null neurons, a C2AB fragment targeted to the presynaptic plasma membrane using a GAP43 domain effectively rescues fast neurotransmission as detected by electrophysiology^18^. Second, Doc2b is more sensitive to Ca^2+^ than Syt1 (refs 25, 26), allowing to perform our measurements at lower Ca^2+^, minimizing the risk of forming Ca^2+^-induced artificial structures previously observed in PS-rich membranes^27^. With this assay, we find that Doc2b triggers the formation of a stable membrane stalk, which allows for lipid but not content mixing. These results show that, *in vitro*, Doc2b C2–phospholipid interactions are sufficient to induce hemifusion in the absence of SNAREs.

## Results

### Doc2b induces the formation of membrane stalks

**Figure 1:**
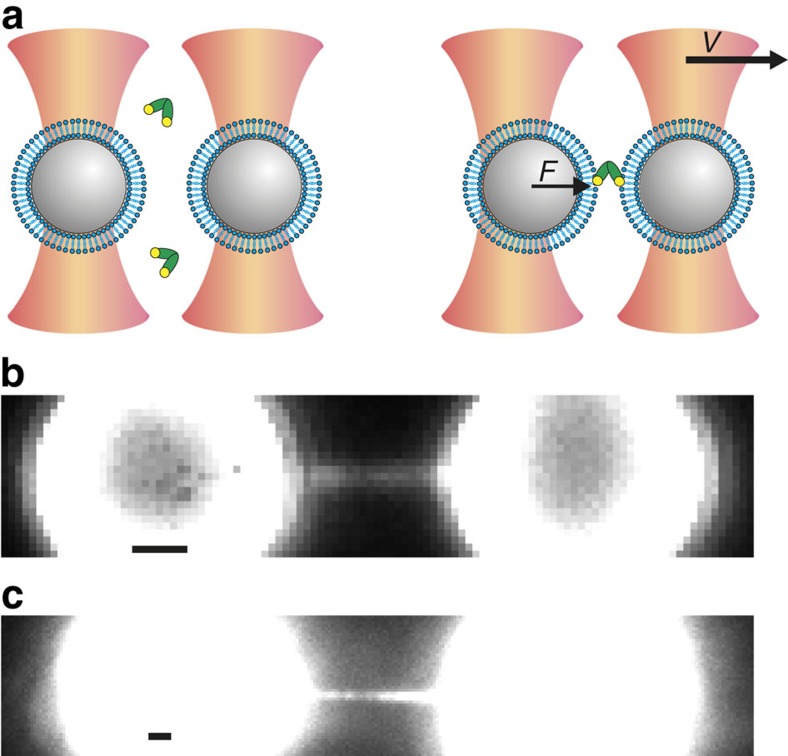
Principle of combined dual-beam optical trapping and fluorescence microscopy to detect membrane fusion. (**a**) Schematic (not to scale) of two polystyrene beads (grey) coated with a lipid bilayer (blue) trapped in focused laser beams (orange). Protein fragments comprising Doc2b (green) were added to the aqueous compartment and could bind phospholipids in the presence of Ca^2+^ (yellow). Upon bead separation (with constant velocity *v*), the force (*F*) on the left bead was measured. Unless otherwise indicated, experiments were performed at 0.74 μM Doc2b with membranes composed of 80% PC and 20% PS and in 250 μM Ca^2+^. (**b**) High-force events were accompanied by the formation of a micrometre-long membrane stalk, revealed by simultaneous fluorescence imaging in the presence of the fluorescent phospholipid NBD-PE. (**c**) Using unlabelled membranes, a fluorescently tagged Doc2b fragment, 0.39 μM C2AB-EGFP, bound efficiently to the entire bilayer including the membrane stalk. Scale bars, 1 μm.

The geometry of fusing membranes was approximated by coating individual microspheres (3.84 μm diameter; Fig. 1a and Supplementary Fig. 1) with phospholipid bilayers containing 20% PS and 80% phosphatidylcholine (PC). Uniform bead coating was confirmed using the fluorescent tracer *N*-(7-nitrobenz-2-oxa-1,3-diazol-4-yl)-1,2-dihexadecanoyl-*sn*-glycero-3-phosphoethanolamine (NBD-PE) in control samples, as well as by electron microscopy where the thickness was close to that of a single bilayer (Supplementary Fig. 2). The microspheres were manipulated with subnanometre precision in two optical traps enabling force detection with 0.1 pN precision^28^. To deal with the existing variation in the diameter of the beads, the point of membrane contact was established empirically for each bead pair by an automated procedure (see Methods section: ‘Approach-and-separation routine' for details). After 5 s of membrane contact in buffer containing 0.74 μM Doc2b and 250 μM Ca^2+^, the beads were separated and membrane stalks were observed (Fig. 1a and Supplementary Fig. 3a). The stalks were visualized by real-time imaging of either NBD-PE (Fig. 1b) or Doc2b (0.39 μM C2AB-enhanced green fluorescent protein (EGFP); Fig. 1c). These experiments show that the membrane including the stalk was completely coated with Doc2b, suggesting that it may help to induce or stabilize the stalk.

### Rupture of the membrane stalk requires forces up to 600 pN

**Figure 2:**
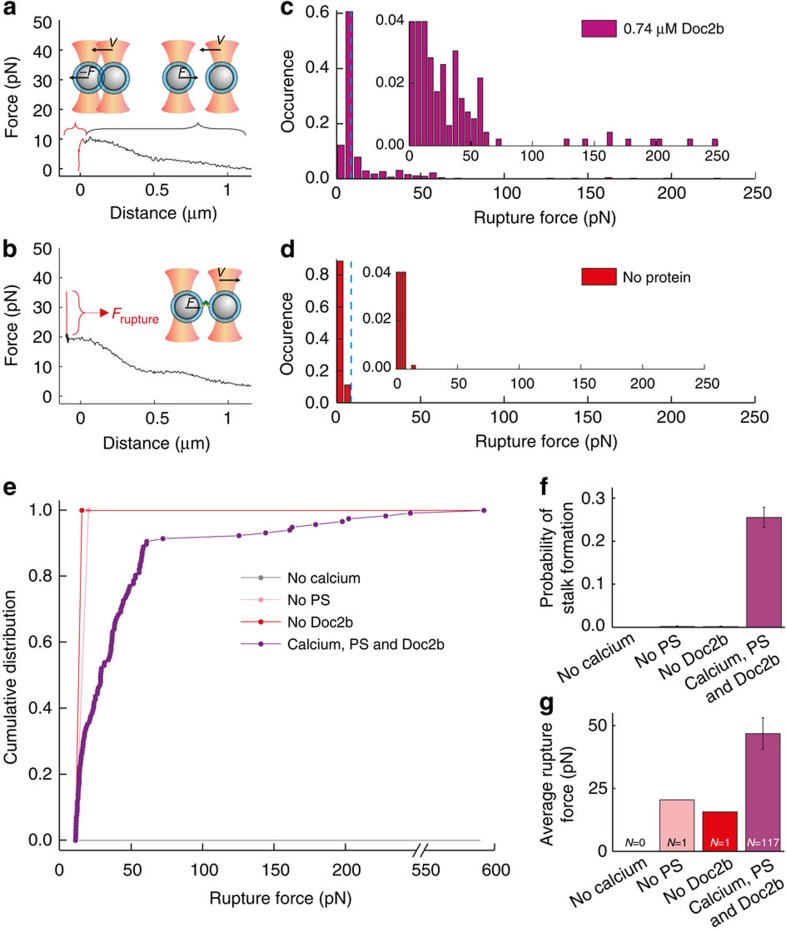
Doc2b induces stalk formation in a Ca^2+^- and PS-dependent manner. (**a**) Typical force–distance curve in case no membrane stalk was formed. During bead approach, the force initially increases because of interference between the two optical traps. When the beads touch, a drop in the force is recorded as the beads are pushed out of their traps. Retraction speed of the bead was 2 μm s^−1^ in all experiments. (**b**) Typical force–distance curve in case of a stalk formation event. A clear peak is observed (shown in red) with a corresponding rupture force (indicated in red as *F*_rupture_) ranging up to 0.6 nN. (**c**) During bead separation in the presence of 20% PS, 250 μM Ca^2+^ and 0.74 μM Doc2b, a broad distribution of rupture forces values was observed (*N*=458). Inset: same distribution but with different scaling of the vertical axis. Blue dashed line shows the threshold of 11.3 pN (defined as mean+4 × s.d., see Supplementary Fig. 4 for details) used to discriminate high-force events. (**d**) In the absence of Doc2b but the presence of 20% PS and 250 μM Ca^2+^, only one high-force event was observed out of 661 trials. Inset as in **c**. (**e**) Cumulative distributions of rupture events in the presence of 0.74 μM Doc2b, 20% PS and 250 μM Ca^2+^ (purple) or in the absence of Ca^2+^ (grey), PS (pink) or Doc2b (red). (**f**) Probability of stalk formation in the absence of Ca^2+^ (0 events during 291 trials), PS (1 event during 556 trials) or Doc2b (1 event during 661 trials) or in the presence of 0.74 μM Doc2b, 20% PS and 250 μM Ca^2+^ (117 events during 458 trials). Error bars indicate the statistical error in the number of counts. (**g**) Corresponding rupture forces (mean±s.e.m.).

To quantitatively study the stalk formation process under various conditions, we hypothesized that it should take significant force to rupture a stalk when separating the beads^29^,^30^. Rupture events can therefore act as readout for stalk formation (Fig. 2a,b and Supplementary Fig. 3). As expected, we observed many interactions between the beads in the presence of all components (Doc2b, Ca^2+^ and PS). The rupture forces of these interactions exhibited a distribution with a prominent peak around 6±1 pN (N=341;±values indicate s.e.m.) and a long tail with events up to 600 pN (*N*=117; Fig. 2c and Supplementary Table 1). In the absence of Doc2b, the rupture forces during bead separation exhibited a distribution with only a peak around 4.18±0.03 pN (*N*=661) and lacking the long tail (Fig. 2d and Supplementary Table 1). Based on this observation we consider this low force peak as the background signal in our experiments and the high force tail as indicative of stalk formation.

To discriminate protein-mediated fusion events from background events, we introduced a threshold defined as the mean+4 times the s.d. of the background signal (Supplementary Fig. 4). We chose this relatively high threshold to make sure that the specific interactions are not overwhelmed by the background (Fig. 2c,d and Supplementary Table 1). The average force of the rupture events in the presence of all components was 46±6 pN in line with previously reported values of membrane stalk rupture^29^,^30^ (Fig. 2g and Supplementary Table 1). Successive approach-retraction cycles did not significantly change the rupture force (Supplementary Fig. 5). The latter is important because it indicates that the lipid coating of the beads remains intact during the experiment.

### Stalk formation requires Ca^2+^ and PS

Using this method for separating signal from background, we tested if the observed stalk formations required Doc2b, Ca^2+^ and PS. High-force ruptures were detected during 117 of 458 bead separations in the presence of Doc2b, Ca^2+^ and PS (26±2%), only in 1 of 661 in the absence of Doc2b (0.2±0.2%), in 0 of 291 in the absence of Ca^2+^ (<0.2%) and in 1 of 556 in the absence of PS in the membrane (0.2±0.2%; Fig. 2e–f and Supplementary Table 1). Thus, the probability of membrane stalk formation increased more than a hundred-fold in the presence of Ca^2+^, PS and Doc2b, indicating that stalk formation is a specific process, which involves C2–phospholipid interactions^31^.

### Membrane hemifusion

**Figure 3:**
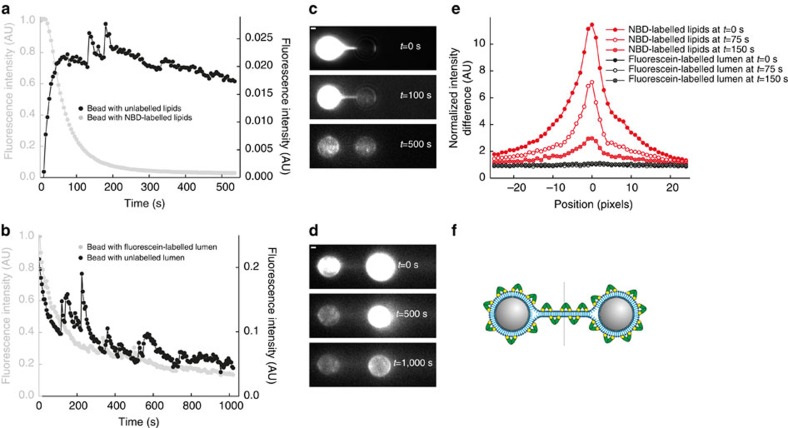
Membrane hemifusion. (**a**) In the presence of fluorescent NBD-PE in a single membrane-coated bead, membrane stalk formation was accompanied by a strong fluorescence increase in the unlabelled membrane and a concurrent decrease in the labelled membrane. This phospholipid mixing indicates that either hemifusion or full membrane fusion occurred. Fluorescence intensities were normalized with respect to the initial intensity on the NBD-labelled bead. Even though stalk formation happens readily, to track lipid continuity and content mixing we require long-lived membrane stalks (>100 s). We measured two of those events. The small peaks in the signal are due to autofluorescent impurities in the buffer flow. (**b**) To distinguish between hemifusion and full fusion, we tested if membrane stalk formation allowed content mixing by coating the beads with nonfluorescent PC/PS liposomes while one of the beads was loaded with fluorescein in the liposomal lumen. On a timescale of 1,000 s, we did not detect a fluorescence increase in the unlabelled bead or membrane stalk, indicative for hemifusion. The decrease in fluorescence of the fluorescein-labelled bead is due to photobleaching. Fluorescence intensities were normalized with respect to the initial intensity on the fluorescein-labelled bead. In total, four of these events were measured. (**c**) Fluorescence images of bead pair in **a** at three different time points. Scale bar, 1 μm. (**d**) Fluorescence images of bead pair in **b** at three different time points. Scale bar, 1 μm. (**e**) Fluorescence intensity profiles along the dashed line indicated in **f** at three different time points. Intensities were averaged over 20 pixels perpendicular to the dashed line and normalized to the background signal. (**f**) Schematic representation of the hemifused configuration with Doc2b bound to the membrane surface.

**Figure 4:**
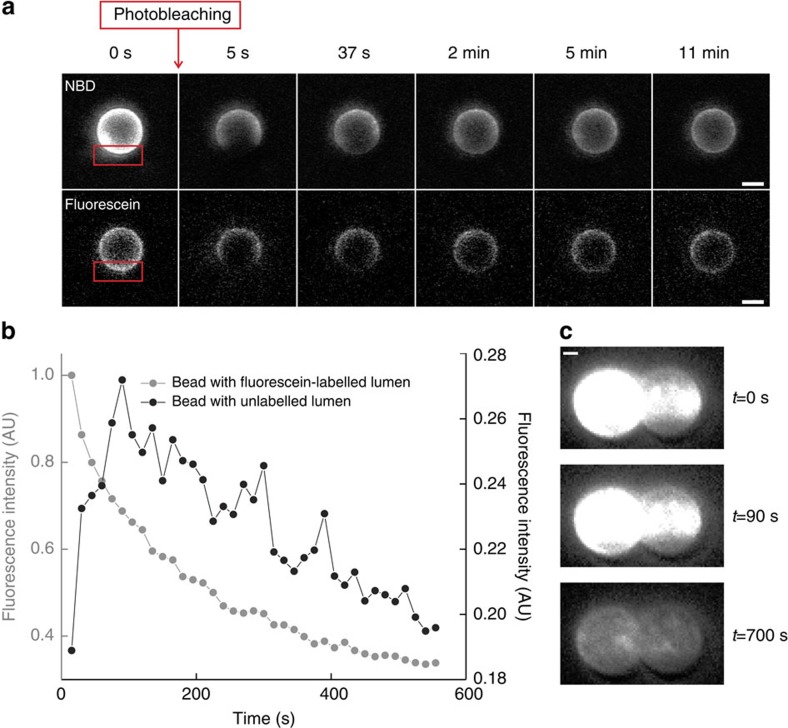
Fluorescent labeling of either lipids or luminal compartment provides a probe for detecting membrane remodeling, hemifusion of fully fused states. (**a**) Membrane-coated beads with lipids labelled with NBD-PE or with fluorescein in the luminal compartment were analysed by confocal microscopy before (*t*=0 s) and after (*t*=5 s) photobleaching of the region indicated by the red rectangle. To assess fluorescence recovery after photobleaching, images were acquired at various times after bleaching. From the fact that the fluorescence intensity recovers after time, we conclude that the fluorophores are free to diffuse through the membrane (in the case of NBD) or within the luminal compartment (for fluorescein). Data shown are representative example from two (in the case of NBD) and ten (in the case of fluorescein) experiments. Scale bar, 2 μm. (**b**) When two beads, of which one has a fluorescein-labelled lumen, are brought in contact in the presence of 5 mM CaCl_2_ in the buffer for at least 1 min, membrane fusion occurred. This fusion was accompanied by an increase of fluorescence signal in the bead that was originally dark (black data set), proving that content mixing, indicative of full membrane fusion, has occurred. The decrease in fluorescence intensity in the grey data set is due to photobleaching. Similar results were obtained in three independent experiments. (**c**) Fluorescence images of bead pair in **b** at three different time points. Note that *t*=0 s is the time at which fluorescence acquisition was started. The initial intensity on the bead (top panel) is thus partially caused by autofluorescence of the beads and partially by content that has already diffused to the other bead. Scale bar, 1 μm.

The Doc2b-covered membrane stalk could represent various molecular configurations: it could consist of (i) two individual membranous tubes connected by a protein bridge; (ii) a structure with a continuous outer membrane leaflet (hemifusion) or (iii) a continuous membrane tube that connects the lumen of the two membrane-coated beads (full fusion). To distinguish between these scenarios, we conducted experiments that probed either lipid or content mixing. To test lipid mixing, we captured one bead coated with 70% PC/20% PS/10% fluorescent NBD-PE and a second bead coated with 80% PC/20% PS. After formation of a membrane stalk, the stalk and unlabelled bead started to fluoresce (Fig. 3a,c,e). Hence, we concluded that the stalk consists of a continuous phospholipid structure as expected for hemifusion or full fusion. To test if the membrane stalk also allows content mixing, we captured a bead with soluble fluorescein, a 389-Da fluorophore small enough to diffuse through a fusion pore, in the luminal compartment between the membrane and bead (Supplementary Fig. 1 for production method), whereas the lumen of the other trapped bead was not fluorescent. Upon Doc2b-induced formation of a membrane stalk, the fluorescence signal of the unlabelled bead and the stalk did not increase (Fig. 3b,d,e). We performed a number of controls to make sure that the absence of fluorescein in the unlabelled bead was not caused by immobilized fluorescein: (i) confocal bead imaging and fluorescence recovery after photobleaching experiments showed that the fluorescein can freely diffuse within the luminal compartment (Fig. 4a) and NBD can freely diffuse through the lipid bilayer (Fig. 4a), and that, upon membrane disruption by 0.02% Triton X-100, the fluorescein leaked into the surrounding buffer (Supplementary Fig. 6); (ii) we showed using Ca^2+^-induced membrane fusion that it is possible to detect content mixing with our method (Fig. 4b,c). Hence, the absence of luminal content mixing we observe in the presence of Doc2b demonstrates that the membrane stalk does not contain a fusion pore. Thus, Doc2b directly induces hemifusion in the absence of SNAREs (Fig. 3f).

**Figure 5:**
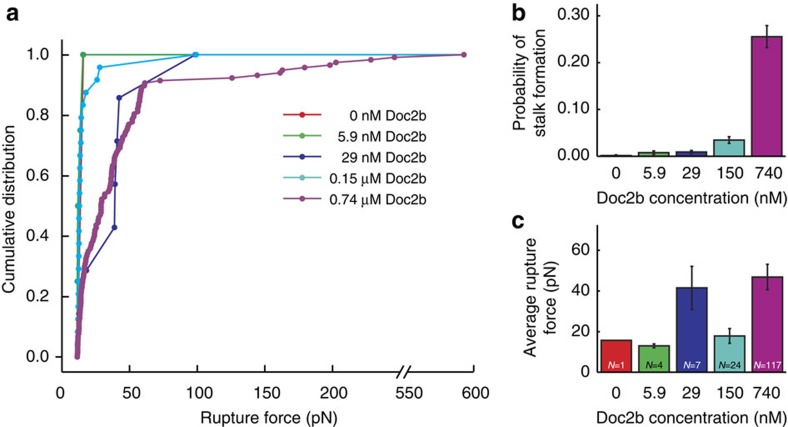
Doc2b concentration dependence of membrane stalk formation. (**a**) Cumulative distributions, (**b**) stalk formation probabilities at 0 nM (1 event during 661 trials), 5.9 nM (4 events out of 521 trials), 29 nM (7 events out of 777 trials), 0.15 μM (24 events out of 694 trials) and 0.74 μM (117 events out of 458 trials) Doc2b. Error bars indicate the statistical error in the number of counts. (**c**) Average±s.e.m. of rupture forces at the same Doc2b concentrations observed during bead separation in the presence of 20% PS and 250 μM Ca^2+^. Numbers of above-threshold events are indicated in the graph. A strong increase in the probability of stalk formation was observed as a function of Doc2b concentration.

To confirm Doc2b dose dependence, rupture force profiles were measured in the presence of 0–0.74 μM Doc2b (Fig. 5a). The hemifusion probability increased drastically from negligible to 26±2% (Fig. 5b and Supplementary Table 1). We could only reliably determine the average rupture for concentrations above 0.15 μM where it increased from 18±4 to 47±6 pN (Fig. 5c and Supplementary Table 1).

## Discussion

The dose-dependent increase in the probability of stalk formation suggests that in our *in vitro* situation, Doc2b binding primarily lowers the energy barrier for hemifusion, whereas the increase in rupture force suggests a stabilization of the membrane stalk due to protein coverage. In previous studies with atomic force microscopy, Syt1–membrane interactions^30^ yielded rupture forces in the similar range of 72−122 pN (at a ∼20 times higher loading rate). Note that in these experiments the molecular arrangement was different because of the immobilization of glutathione *S*-transferase (GST)-C2AB on a cantilever. In another study, atomic force microscopy tips were coated with lipid bilayers to study Synapsin-1–mediated interactions between opposing membranes^29^. At a retraction velocity of 80–400 nm s^−1^, this yielded rupture forces up to 3 nN, which corresponds well with our highest observed force of 0.6 nN.

To conclude, our data suggest that Doc2b by itself can promote hemifusion in a manner that depends on Ca^2+^-dependent binding to PS in the membrane. This activity likely relates to the phenomenon that C2 domain insertion induces membrane curvature, as was previously shown for the C2AB fragments of Syt1 and Doc2b, which caused liposome tubulation in cell-free preparations^17^,^25^,^32^. In turn, membrane curvature is predicted to strongly affect the energy state of the hemifusion intermediate according to theoretical models of stalk formation^33^. Together, this suggests that Doc2b contributes to Ca^2+^-dependent secretion by lowering the energy barrier of hemifusion through direct interaction with PS. This finding may also shed light on an open question regarding the mechanism of Doc2b-dependent enhancement of spontaneous neurotransmitter release in neurons^25^,^34^. Although our *in vitro* data suggest a direct Ca^2+^ sensing role by Doc2b in membrane fusion, the mechanism in living systems is likely more complex and additional interactions of Doc2b with SNARE proteins^25^, Munc18 (ref. 35) and Munc13 (ref. 36) must also be considered. In support of this idea, mutations in Doc2b that inhibit SNARE binding, inhibit the fusogenic activity of SNARE-containing liposomes partially (but not completely)^25^. As C2AB domains and their phospholipid interaction surfaces are highly conserved between Ca^2+^-sensors, a similar activity is likely to regulate many secretory processes in the body.

Finally, our method may aid the discovery of membrane fusion mechanisms. Liposome fusion assays have yielded tremendous insights into SNARE-mediated membrane fusion^9^ but do not allow detection of the SNARE-independent effects of Ca^2+^ (ref. 37). Our unique combination of force spectroscopy with fluorescence microscopy enables both direct quantification and visualization of fusion events, while at the same time providing direct evidence for possible lipid and content mixing.

## Methods

### Bead coating

1,2-Dioleoyl-*sn*-glycero-3-phosphocholine and 1,2-dioleoyl-*sn*-glycero-3-phospho-L-serine were acquired from Avanti Polar Lipids as solutions in chloroform. NBD-PE was acquired from Invitrogen as chlorophorm solution. The protocol for coating beads with lipids was adapted from previous work^38^. Polystyrene nonporous beads of diameter 3.84 μm±4% (manufacturer's specifications) were acquired from Spherotech. For each coating procedure, the beads were washed three times in milliQ and sonicated shortly. Liposomes were prepared by mixing the lipids from chloroform stocks in desired proportion, extensively drying the mix under a nitrogen gas stream, and reconstituting the lipids in milliQ to a final concentration of 1 mg ml^−1^. The solution was vortexed to form giant multilamellar vesicles and then sonicated on ice (8 times for 6 s, with at least 1 min interval between each sonication) to produce small unilamellar vesicles. To get rid of the residual multilamellar vesicles, the solution was centrifuged at 21,000*g* for 30 min at 4 °C. The supernatant was mixed with beads, with addition of CaCl_2_ to 3 mM, and incubated for 16 h at 4 °C with gentle continuous mixing to keep the beads in suspension. The beads were washed (by spinning at 900 g and gently resuspending) in buffer 1 (25 mM HEPES, pH 7.4, 200 mM NaCl, 1 mM tris(2-carboxyethyl)phosphine (TCEP), 5 mM EDTA), then buffer 2 (25 mM HEPES, pH 7.4, 100 mM NaCl, 1 mM TCEP, 0.25 mM CaCl_2_) and then in buffer 3 (25 mM HEPES, pH 7.4, 25 mM NaCl, 1 mM TCEP and 0.25 mM CaCl_2_). The coated beads were stored at 4 °C and used within 5 days.

To monitor the bead-coating procedure, we prepared in parallel a sample with addition of 1.5% fluorescent NBD-PE. These beads were imaged in solution by confocal microscopy on a LSM510 meta system (Carl Zeiss) with excitation at 488 nm (emission 514 nm) with a × 63 oil objective (numerical aperture=1.3). In addition, we prepared a batch of beads containing 10% NBD-PE in the membrane coating for the imaging experiments (Figs 1b, 3a,c,e and 4a and Supplementary Figs 2a and 6). As stalk formation was observed with (Fig. 1b) and without NBD-PE (Fig. 1c) and similar rupture forces were measured under both conditions, we concluded that the addition of NBD-PE does not have a significant effect on stalk formation. For the experiment shown in Figures 3b,d,e and 4 and Supplementary Fig. 6, we used fluorescein-containing beads. To prepare these beads, the procedure was the same as above except that the dried phospholipids were suspended in a solution containing 250 μM fluorescein-5-isothiocyanate (Molecular Probes) dissolved in 10 mM Tris, pH 7.4, leading to the inclusion of fluorescein in the lumen of the liposomes and, ultimately, of the membrane-covered beads (see Supplementary Fig. 1 for details).

### Recombinant proteins

Recombinant Doc2b C2AB (rat, amino-acid residues 115–412) was subcloned into the pGEX vector (GE Healthcare), and transformed into *E. coli* BL21 DE3 strain for expression. To express a fusion protein of Doc2b aa115–412 and EGFP, the 231-bp *Eco*R1-*Not*I fragment of pGEX-GS-C2AB was replaced with the 989-bp *Eco*R1-*Not*I fragment of pEGFP-N2-Doc2b (ref. 39). Cultures in LB medium supplemented with 200 μg ml^−1^ ampicillin were grown to OD_600_=0.6–0.8 and gene expression was induced with 0.5 mM IPTG at 37 °C for 4 h. Bacterial pellets were lysed in 300 mM NaCl, 50 mM Tris, pH 7.5, 10 mM EDTA, 1 mg ml^−1^ lysozyme and protease inhibitor cocktail, sonicated five times for 15 s, incubated in the presence of 1% Triton X-100 and centrifuged for 30 min at 10,000*g* to remove insoluble cell debris. GST-tagged C2 domains were isolated from bacterial lysates by affinity purification with glutathione agarose (Sigma), washed extensively in high-salt buffer (300 mM NaCl, 50 mM Tris pH 7.4, 10 mM EDTA). The beads were then loaded into a disposable column where the buffer was replaced with DNA/RNA digestion buffer (150 mM NaCl, 50 mM Tris pH 7.4 and 1.4 mM MgCl_2_), followed by incubation with 1 mg ml^−1^ each of DNAseI (Roche) and RNAse A (Boehringer Mannheim) for 20 min at 37 °C. The beads were washed again five times with high-salt buffer and three times with low-salt buffer (150 mM NaCl and 50 mM Tris, pH 7.4). C2AB domains were then cleaved from GST using thrombin (Serva) for 16 h at 4 °C. Protein concentration was determined by SDS–PAGE and SYPRO Ruby staining, using bovine serum albumin (Sigma) as a standard. Proteins were diluted to the indicated concentration in buffer 3 as specified above. All buffers were filtered through a 0.2-μm pore size and stored at 4 °C for no longer than a week.

### Optical trapping

All measurements were performed at room temperature. The optical tweezers setup used was previously described in detail^40^. Briefly, the combined optical trapping and fluorescence-microscopy setup was custom-built around a Nikon inverted microscope, equipped with a 1,064-nm trapping laser. The laser beam is split into two perpendicularly polarized beams using a half-wave plate and a polarizing beam splitter to produce two independent traps that can be used to manipulate two individual beads. The position of the first trap can be controlled by steering one of the telescope lenses. The two beams are combined using another polarizing beam splitter and coupled into a water-immersion objective. Force-detection on one of the beads is then achieved by collecting the transmitted light in an oil-immersion condenser, rejecting the unwanted polarization using a polarizing beam splitter and imaging on a position-sensitive diode.

Using a blue light-emitting diode to illuminate the sample, bright-field images of the trapped beads were obtained on a CCD camera allowing real-time determination of the bead-to-bead distance. For the wide-field fluorescence microscopy, NBD-PE, EGFP and fluorescein were excited by a 491-nm laser, coupled into the microscope and imaged on an EMCCD camera. A custom-made glass multichannel laminar flow cell was used to obtain parallel flow and to facilitate rapid buffer exchange between channels containing lipid-coated beads, buffer and protein.

### Approach-and-separation routine

When the beads were kept far apart (>2 μm), the measured force was zero. When the beads were moved very close together (<0.5 μm) one optical trap exerted a force on the bead in the other trap in the range of 5–15 pN (see Fig. 2a for details); an effect that was independent of lipid coating on the beads. Because of the intrinsic variation in bead size (standard deviation 4% as indicated by the manufacturer), an automated stepping procedure was employed for each bead pair to determine the distance at which specific Doc2b-induced interactions occurred (Supplementary Fig. 3).This stepwise exploration was stopped as soon as a rupture force of at least 5 pN was detected during bead separation. The approach-and-retraction cycle was then repeated 20–160 times using the same distance (Supplementary Fig. 3b, arrows indicate rupture events). During many repeated cycles there was no detectable trend in the observed force values, suggesting that the membrane coating remained intact (Supplementary Fig. 5). The speed at which the bead is retracted is known to influence rupture forces. Therefore, a constant trap speed of 2 μm s^−1^ was used in all experiments.

### Data acquisition and analysis

Data analysis was performed using custom-written software in LabView (National Instruments) to extract force data at a frequency of 50 kHz. In post-processing, peak heights were determined from force traces (such as Supplementary Fig. 3b) by subtracting the baseline (average of force-values before the peak) from the maximum force (at the top of the peak). To discriminate between stalk formation events and unsuccessful events, we used a single threshold, determined by performing a Gaussian fit to the major peak in the rupture force distribution containing all observed peaks (Supplementary Fig. 4). We then used the mean value plus four times the standard deviation of this peak as a threshold, resulting in a threshold value of 11.3 pN.

### Electron microscopy

Polystyrene beads were coated with lipid membrane as described above, and incubated with protein and Ca^2+^-containing buffers as indicated. The beads were fixed and contrasted by 1% osmium-tetraoxide (EMS) before embedding in 10% gelatine droplets for cryo-sectioning. 70 nm sections were collected on carbon/formvar-coated mesh grids and stabilized by methylcellulose in the presence of 0.4% uranyl acetate when indicated. Transmission electron microscopy images were collected on a JEOL1010 TEM (JEOL) with Morada CCD camera (Olympus Soft Imaging Solutions) at × 80,000 magnification. The thickness of the electron-dense coating was quantitated in iTEM software (Olympus).

## Additional information

**How to cite this article:** Brouwer, I. *et al.* Direct quantitative detection of Doc2b-induced hemifusion in optically trapped membranes. *Nat. Commun.* 6:8387 doi: 10.1038/ncomms9387 (2015).

## Supplementary Material

Supplementary Information Supplementary Figures 1-6 and Supplementary Table 1
