# Supplementary material for: Direct quantitative detection of Doc2b-induced hemifusion in optically trapped membranes
Source: Nat Commun. 2015 Sep 23;6:8387. doi: 10.1038/ncomms9387 (PMC4598560; doi:10.1038/ncomms9387)
Supplement: Supplementary Information — Supplementary Figures 1-6 and Supplementary Table 1 [file ncomms9387-s1.pdf]

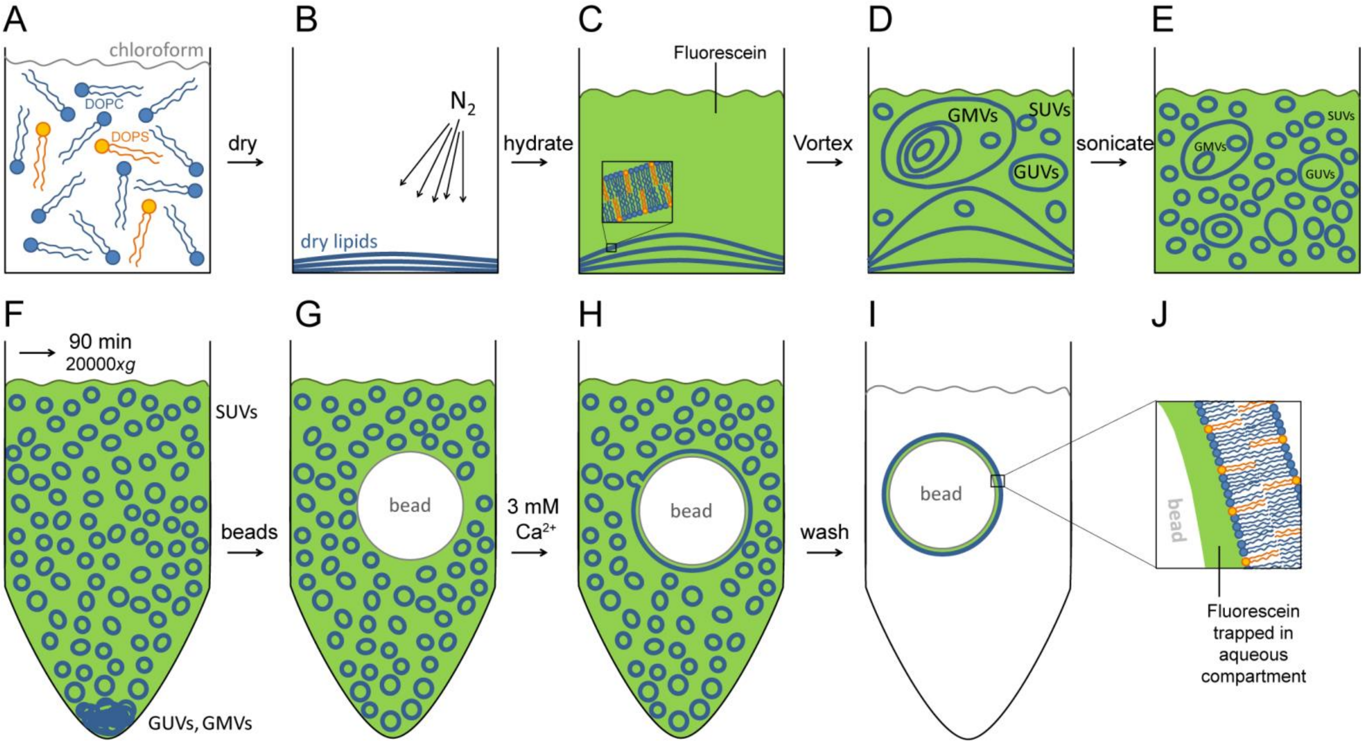

Supplementary Figure 1. Method for preparation of lipid-coated beads and fluorescein uptake into the aqueous compartment of membrane-covered beads. (A) Phospholipids dissolved in chloroform were mixed to obtain the desired mixture (80% DOPC and 20% DOPS) in a glass container. (B) The phospholipid mixture was dried under a nitrogen stream. (C) The dried lipids were hydrated in H2O containing 10 mM Tris pH 7.4 and 250 µM fluorescein. For the beads without fluorescent lumen, the fluorescein was left out of the hydration mix, further preparation method was identical. (D) The mixture was Vortexed for 1 min to obtain a suspension containing smaller membranous structures including giant multilamellar vesicles (GMVs), giant unilamellar vesicles (GUVs) and small unilamellar vesicles (SUVs). (E) The mixture was sonicated on ice to disrupt larger structures and produce more SUVs (liposomes). (F) The mixture was tranferred to an Eppendorf container and larger structures were removed by centrifugation for 90 min at 20,000 x g. (G) The supernatant was transferred to a new Eppendorf tube and polystyrene beads (3.84 µm in diameter) were added. This schematic only depicts a single bead for illustrative purposes. (H) $CaCl_2$ was added to a final concentration of 3 mM, inducing fusion of the liposomes onto the bead during incubation for 16h or longer at 4°C. (I) The membrane-covered beads were washed three times, first in buffer 1 (Hepes pH 7.4, 200 mM NaCl, 1 mM TCEP, 5 mM EDTA), then buffer 2 (25 mM Hepes pH 7.4, 100 mM NaCl, 1 mM TCEP, 0.25 mM CaCl2) and then in buffer 3 (25 mM Hepes pH 7.4, 25 mM NaCl, 1 mM TCEP and 0.25 mM CaCl2). After each wash, the beads were collected by spinning at 900 g for 3 min and gently resuspending the beads. (J) Conceptual drawing of the membrane-covered bead surface with fluorescein retained in the aqueous compartment.

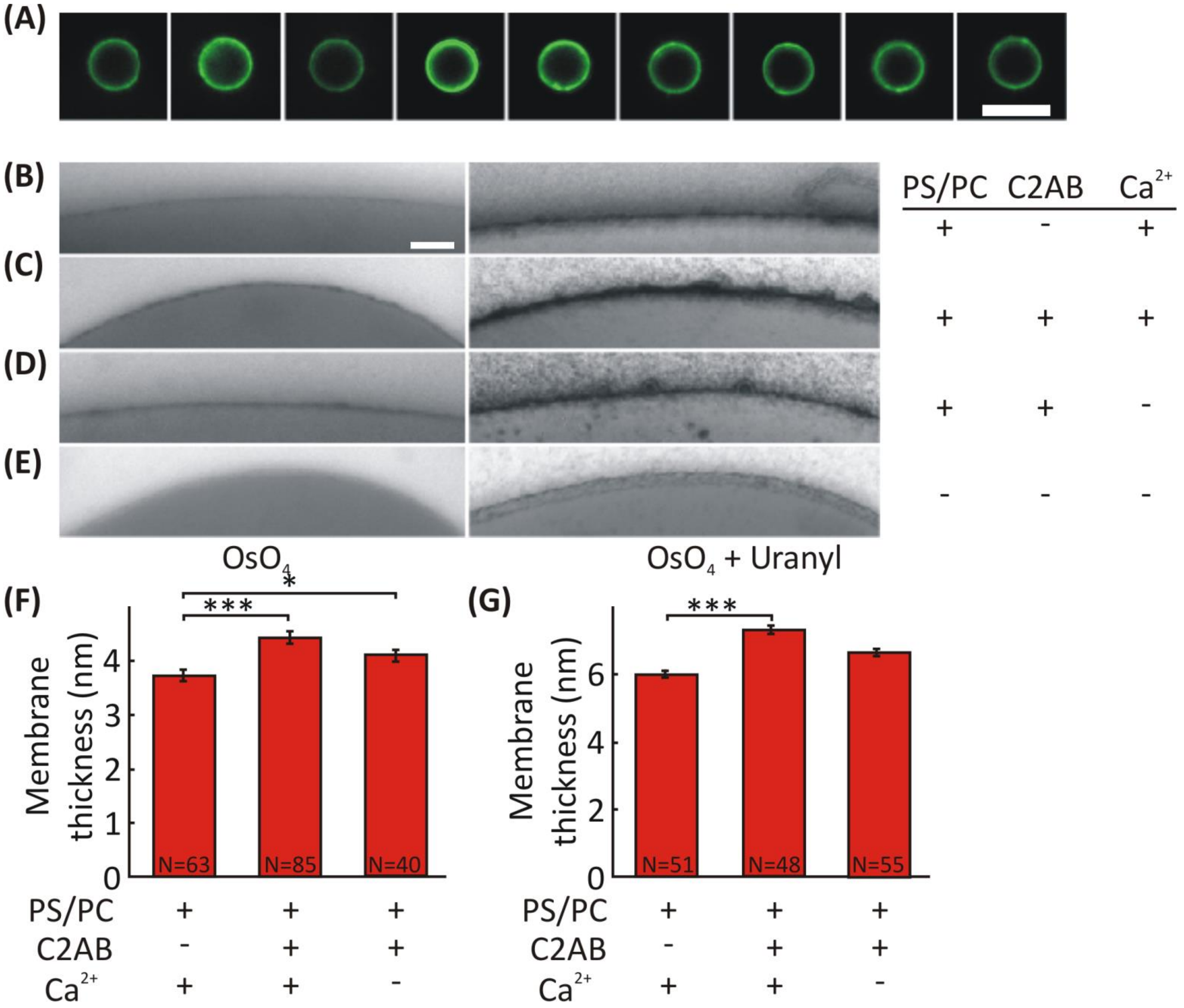

Supplementary Figure 2. Liposome absorption to polystyrene beads results in the formation of a uniform phospholipid bilayer. (A) Typical examples of beads covered with 75%PC, 20% PS and 5% NBD-PE. Confocal imaging shows a complete coverage of the bead (3.84 µm in diameter) with the fluorescent phospholipid NBD-PE. Scalebar: 5 µm. (B)-(E) Electron microscopy images of polystyrene beads in presence or absence of phospholipids (80%PC/20%PS), protein (0.74 µM Doc2b) and Ca^{2+} (250 µM) as indicated on the right. In the left column, OsO_4 was applied as contrasting agent to stain lipids only. Note the presence of a well-defined electron-dense layer except in absence of phospholipids (E), where the bead produces some background staining but lacks an electron-dense layer. In the right column, both OsO_4 and uranyl were applied as contrasting agents to stain lipids combined with proteins. Under these conditions the bead surface acquires some contrast even in absence of phospholipdis and protein (bottom). In presence of phospholipids and/or protein, the membrane coverage was clearly visible as a thin electron-dense layer covering the entire bead surface. Scalebar: 100 nm. (F)-(G) The thickness of the electron-dense layer ±sem, derived from electron micrographs after staining with OsO_4 alone (F) or both OsO_4 and uranyl (G). For comparison the thickness of a single phospholipid bilayer is known to be 4 nm. ***, $p<0.001$; *, $p<0.05$, Mann-Whitney test.

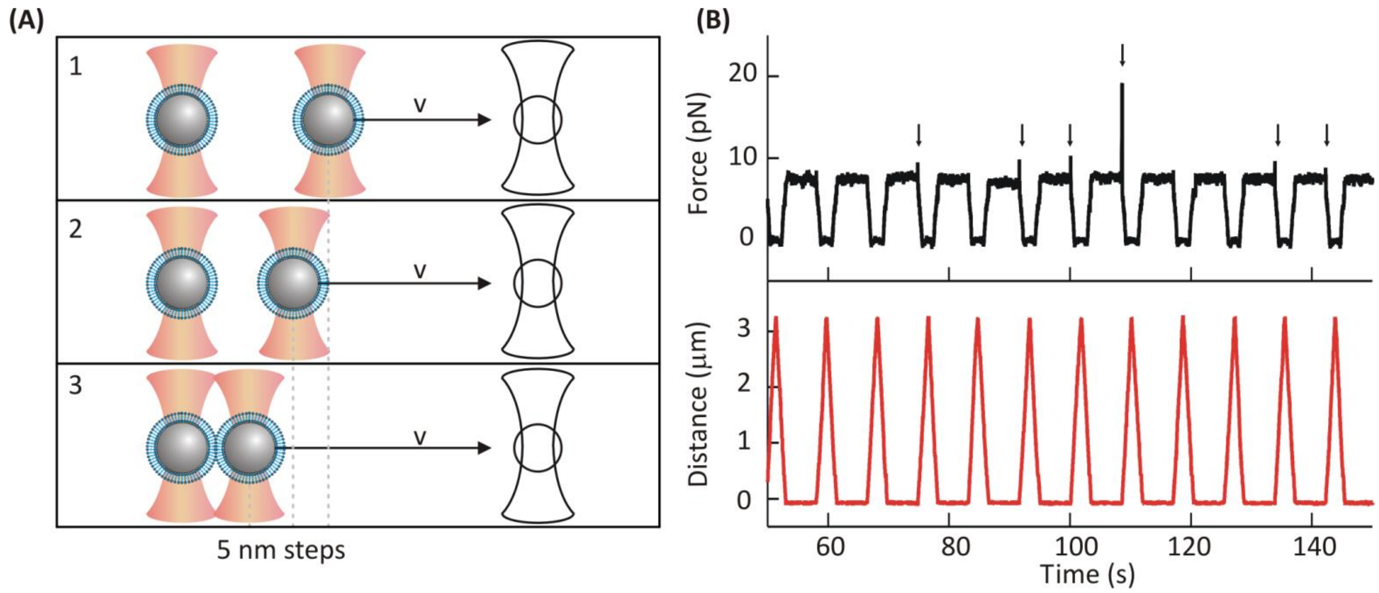

Supplementary Figure 3. Stepping Algorithm used to probe successive membrane stalk formation events. (A) Schematic representation of the stepping algorithm. Between consecutive probing events, the distance between the beads was decreased in 5 nm steps. After 5s to allow for interactions to occur, one bead was retracted at a constant speed of 2.0 µm/s. If the bead separation was accompanied by significant force increase, the stepping algorithm was stopped and the same bead distance was probed repeatedly. (B) Force and distance signals recorded over time during multiple approaches and retractions of the right bead. Occasionally, high force peaks occurred, such as visible indicated by arrows, suggesting the rupture of a structure connecting the beads.

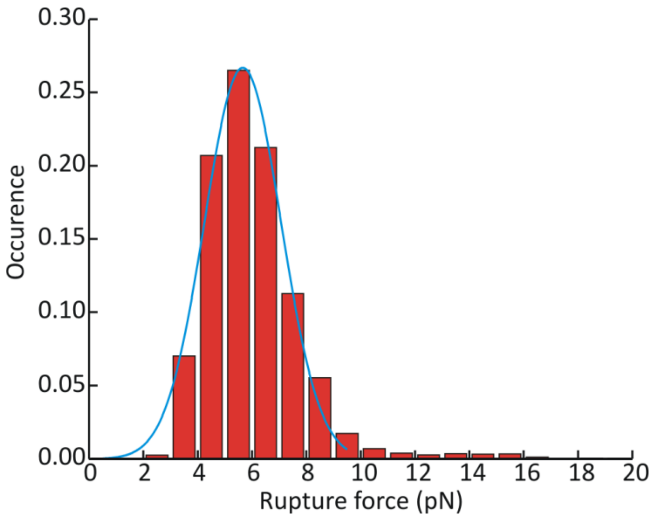

Supplementary Figure 4. Histogram of peak forces and definition of a threshold to detect membrane stalk formation events. A Gaussian fit (blue line) was performed on the main peak in the histogram of recorded force peaks during all 5800 bead separations (red bars), determining a mean value of 5.7 pN and a standard deviation of 1.4 pN. To discriminate membrane stalk formation events, a threshold was defined as mean + 4 SD, which equals 11.3 pN. Bead separations with peak forces below this value were excluded from the analysis shown in Figs 2e-g and 5.

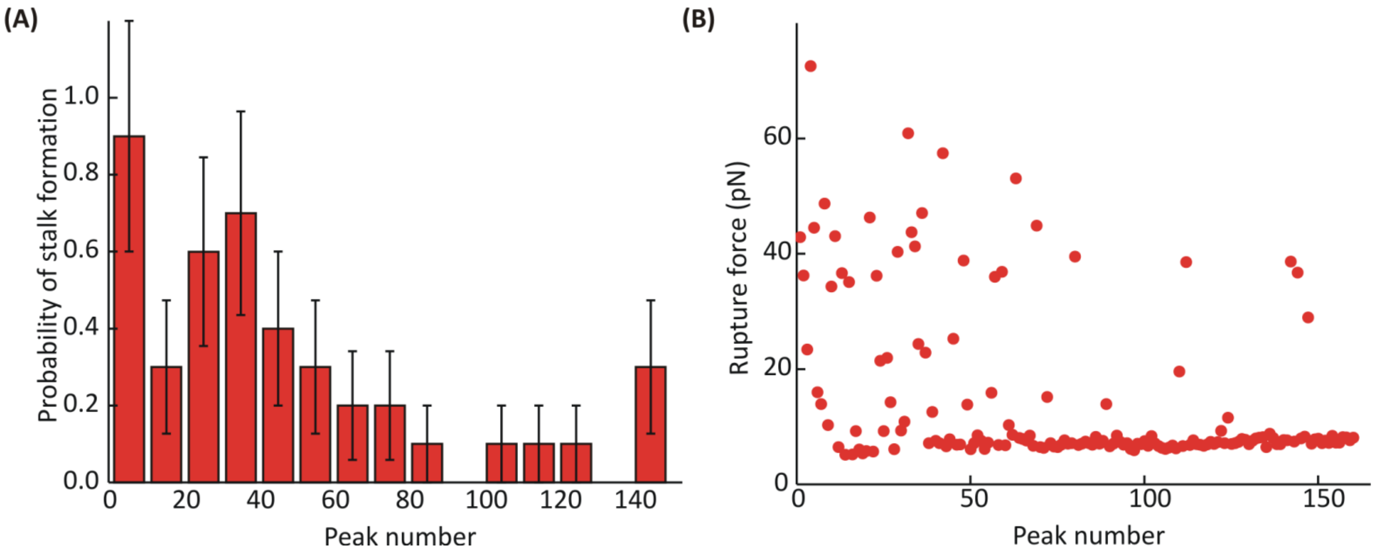

Supplementary Figure 5. Lipid coating remains intact during force measurements. (A) Stalk formation probabilities (average ± sem of 10 successive peaks) during measurements with the same bead pair and (B) successive rupture force values obtained with the same bead pair during repeated measurements. Values do not change significantly, showing that the lipid coating remains intact during the entire experiment.

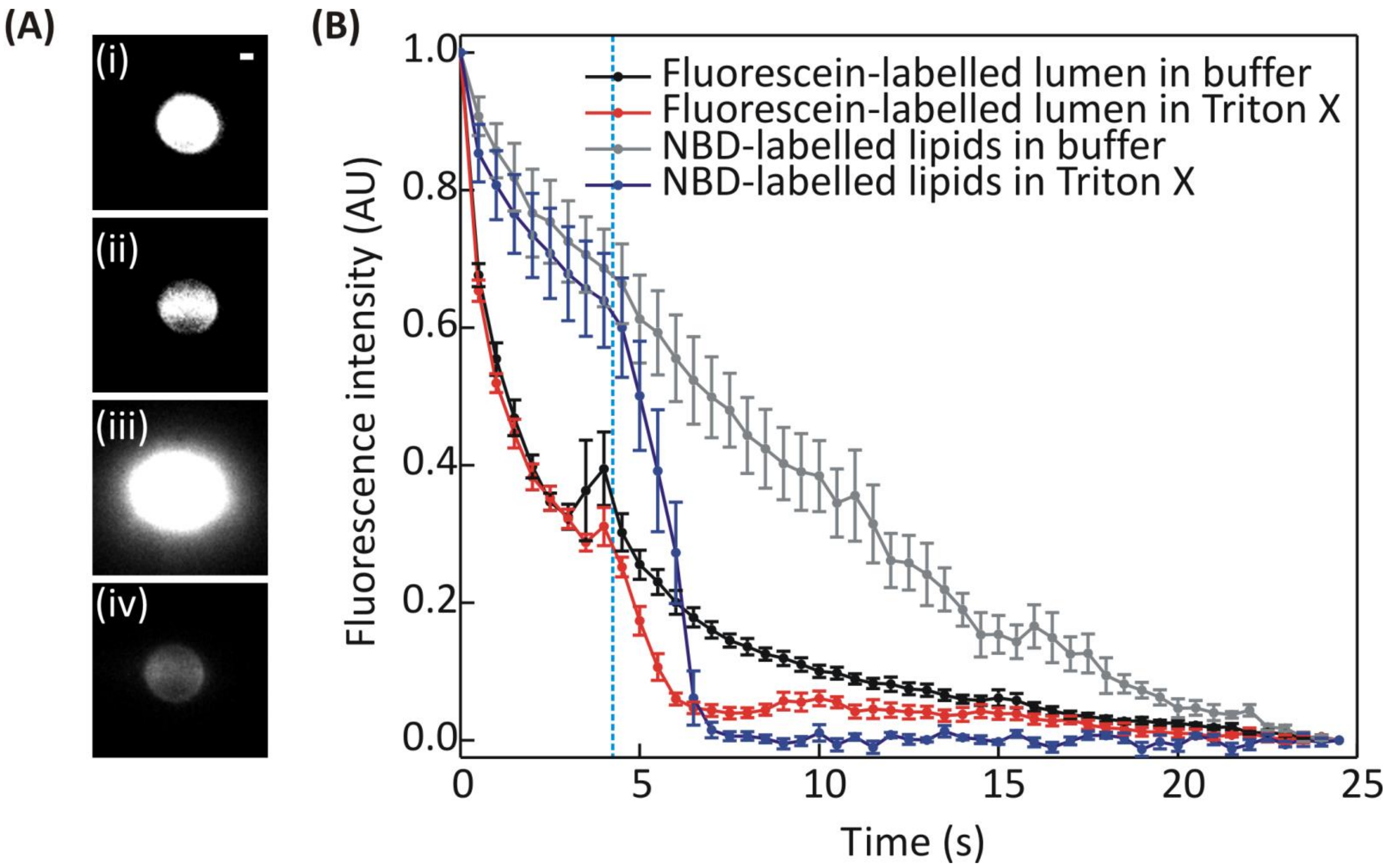

Supplementary Figure 6. Fluorescein encapsulated in the luminal compartment and NBD-labeled lipids detached from the bead surface after detergent exposure. (A) Fluorescence imaging of optically trapped beads containing fluorescein before (i) and after (ii) rupture of the membrane by exposure to 0.02% Triton X-100. Same for NBD-labeled lipids ((iii) and (iv) respectively). (B) Normalized fluorescence intensity over time for fluorescein- and NBD-labeled beads in the buffer channel and in a channel containing 0.02% Triton X-100. Traces shown are averages ± sem of 10 (for black and red dataset), 12 (for grey dataset) and 15 (for blue dataset) individual traces. At timepoint indicated by the blue dashed line the beads entered the channel containing either Triton X-100 (for red and blue datasets) or the buffer channel (for black and grey datasets). On top of exponential decay observed in all traces due to photobleaching, a sudden drop in in intensity was observed around 5-8 s in presence of Triton X-100. This indicates membrane rupture and suggests that both fluorescein and NBD were mobile.

**Lipid composition**	**Ca^{2+} concentration (µM)**	**Doc2b concentration**	**Number of attempts**	**Number of stalk formation events**	**Probility of stalk formation**	**Average rupture force (pN)**
20% PS/80 % PC	250	0 nM	661	1	0.002±0.002	16±16
20% PS/80 % PC	250	0.74 µM	458	117	0.26±0.02	47±6
20% PS/80 % PC	0	0.74 µM	291	0	NaN	NaN
100 % PC	250	0.74 µM	556	1	0.002±0.002	20±20
20% PS/80 % PC	250	5.9 nM	521	4	0.008±0.004	13±1
20% PS/80 % PC	250	29 nM	777	7	0.009±0.003	41±11
20% PS/80 % PC	250	0.15 µM	694	24	0.035±0.007	18±4

Supplementary Table 1. Total number and probabilities (error bars indicate the statistical error in the number of counts) of attempted and successfull stalk formation events and corresponding average rupture forces (± sem).
